# EXERCISE SLOWS NEURAL STEM CELL AGING AND AD RISK WITH CLONAL SELECTION

**DOI:** 10.1093/geroni/igac059.2912

**Published:** 2022-12-20

**Authors:** Wang Xiang, Lei Peng, Maxwell Bay, Michelle Liu, Jonathan Levi, Tianli Ding, Fifi Pan, Michael Bonaguidi

**Affiliations:** University of Southern California, Los Angeles, California, United States; University of Southern California, Los Angeles, California, United States; University of Southern California, Los Angeles, California, United States; University of Southern California, Los Angeles, California, United States; University of Southern California, Los Angeles, California, United States; University of Southern California, Los Angeles, California, United States; University of Southern California, Los Angeles, California, United States; University of Southern California, Los Angeles, California, United States

## Abstract

Exercise acts as a countermeasure against aging and Alzheimer’s disease (AD). Several lines of evidence indicate that the beneficial effects of exercise target neurogenesis and synaptic plasticity. However, how exercise shapes neural stem cell (NSC) function to maintain lifelong hippocampal plasticity remains unknown. Here, we utilize single-cell technologies to uncover exercise slows NSC aging and reduces AD risk. In vivo single-cell lineage tracing revealed that exercise promotes clonal selection by activating quiescent NSCs for self-renewal and depleting neurogenic NSCs. Interestingly, NSC symmetric self-renewal compensated for those lost to differentiation allowing for enhanced neurogenesis without prematurely depleting the overall NSC pool. Prolonged clonal tracing further showed that the selected self-renewed NSCs are more resistant to aging-related NSC depletion. Single-cell RNA-sequencing and multiple bioinformatic analyses determined that exercise slows NSC molecular aging. In particular, exercise mitigates an age-associated increase of AD risk gene expression within NSCs. Our results demonstrate a new regenerative function of exercise to promote healthier brain aging.

